# Preparation, Optimization, and Evaluation of Epichlorohydrin Cross-Linked Enset (*Ensete ventricosum (Welw.) Cheeseman*) Starch as Drug Release Sustaining Excipient in Microsphere Formulation

**DOI:** 10.1155/2020/2147971

**Published:** 2020-04-04

**Authors:** Desta Tesfay, Solomon Abrha, Zewdu Yilma, Gebremariam Woldu, Fantahun Molla

**Affiliations:** ^1^Department of Pharmacy, College of Medicine and Health Sciences, Adigrat University, P.O. Box 50, Adigrat, Ethiopia; ^2^Department of Pharmaceutics, School of Pharmacy, College of Health Sciences, Mekelle University, P.O. Box 1871, Mekelle, Ethiopia; ^3^Department of Pharmacy, College of Medicine and Health Sciences, Bahir Dar University, P.O. Box 79, Bahir Dar, Ethiopia; ^4^Department of Pharmacy, College of Health Sciences, Axum University, P.O. Box 1010, Axum, Ethiopia

## Abstract

*Ensete ventricosum* (Welw.) cheeseman which belongs to the family of *Musaceae* is one of the main sources of starch in Ethiopia. This study aimed at evaluating epichlorohydrin cross-linked enset starch as a drug release sustaining excipient in microsphere formulations of theophylline. Extracted enset starch was cross-linked using epichlorohydrin as a cross-linking agent. The effect of cross-linker concentration, cross-linking duration, and cross-linking temperature on the degree of cross-linking and release rate of microspheres prepared by emulsion solvent evaporation method was investigated using the two-level full factorial design. Accordingly, the concentration of epichlorohydrin and duration of cross-linking were the most significant factors affecting both the degree of cross-linking and drug release rate. Thus, the effects of these two factors were further studied and optimized using the central composite design. As per the numerical method of central composite design, the optimal points were obtained at epichlorohydrin concentration of 13.70% and cross-linking time of 3.82 h. Under these optimal conditions, the model predicts the degree of cross-linking of 74.70% and drug release rate of 28.00 h^1/2^. The validity of these optimal points was confirmed experimentally. The microspheres of the optimum formulation also exhibited minimum burst release with sustained release for 12 h. Besides, the optimized formulation followed the Higuchi square root kinetic model with non-Fickian diffusion release mechanism. The finding of this study suggested that cross-linked enset starch can be used as an alternative drug-release-sustaining pharmaceutical excipient in microsphere formulation.

## 1. Introduction

Starch is an abundant, inexpensive, naturally renewable, and a major reserve polysaccharide of plant sources. It is used as a source of energy during periods of dormancy and regrowth [1]. Of its wide applications in diverse areas of polymer science, starch has been used successfully as a polymer particularly in the packaging industry and in the pharmaceutical manufacturing [2].

As native starch has poor physicochemical properties and several limitations such as poor flow property, the use of starch in pharmaceutical manufacturing often require some form of modifications. Different methods have been used to modify the functional characteristics of native starches in a way that can boost its pharmaceutical applications. Amongst the different techniques for starch modification, chemical and physical methods are the most common ones [1, 3].

Due to the ease of chemical reaction, a wide range of possible applications or versatile function of cross-linked starch, low cost of reaction and their safety, chemical modification in general, and cross-linking in particular is becoming the most widely used method of starch modification in the starch industry [4, 5]. Cross-linking is performed by treating granular starch with bifunctional or multifunctional reagents that are capable of forming either ether or ester linkages with hydroxyl groups in the starch with the tendency to alter the thermal transition characteristics, viscosity, swelling, solubility, and water sorption property of the starch [6, 7].

*Ensete ventricosum* (Welw.) cheeseman, which belongs to the family of *Musaceae*, is one of the main sources of starch in Ethiopia, particularly in the southern part. It resembles a large, thick, single-stemmed banana plant [8].The application of native starches to control the release of therapeutic agents has been reported to be discouraging, and that was mainly attributed to the poor physicochemical properties (that have an effect on the release) of therapeutic agents [9].

Numerous reports pointed out that cross-linking of native starch have the tendency to increase the viscosity, decrease swelling, solubility, and water sorption property of the starch. These improved properties suggest the potential application of cross-linked enset starch as a sustained release pharmaceutical excipient [6, 9, 10]. Thus, the aim of the present work was to prepare cross-linked enset starch and evaluate its potential application as a sustained release excipient in microsphere formulation using theophylline as a model drug for sustained release of active ingredients.

Microsphere is a system in which the drug substance is either homogenously dissolved or dispersed in a polymeric matrix and show different release properties compared to microcapsules. Microspheres/microparticles constitute an important subset of drug delivery systems by virtue of their small size and efficient carrier characteristics [11].

## 2. Materials and Methods

Fresh enset starch the so called “bulla” was purchased from the local farmers around Wolkite, Gurage Zone in the southern part of Ethiopia. Epichlorohydrin (FINAR® Chemicals Ltd., Ahmedabad, India), sodium metabisulphite (Guangzhou Jinhaunda Chemical Reagent Co. Ltd., Guangzhou, China), cyclohexane (UNI-CHEM®, Chemical reagents, India), span-80 (UNI-CHEM®, Chemical reagents, India), chloroform (UNI-CHEM®, Chemical reagent, India), potassium dihydrogen orthophosphate (LOBA Chemie Pvt. Ltd., Mumbai, India), and disodium hydrogen orthophosphate (TITAN BIOTECH LTD., Rajasthan, India) were purchased from local markets in Ethiopia. Anhydrous theophylline (Shandong Xinhua Pharmaceutical Co. Ltd., Zibo, China) was donated by the Addis Pharmaceutical Factory (APF) Pvt. Ltd. Co. All chemicals were of analytical grade and were used as received.

### 2.1. Isolation of Starch from Enset (*Enset Ventricosum* (Welw.) Cheeseman) Plant

To extract starch, first, bulla was soaked in large quantities of distilled water containing 0.075% (*w*/*v*) of sodium metabisulphite. The soaked material was allowed to settle, and the supernatant was decanted. The sedimented starch was then washed repeatedly with the sodium metabisulphite solution until the suspension became clear. The material was passed through a fine muslin cloth to remove cell debris, and the clear suspension was collected and filtered through a fine sieve (224 *μ*m) and was allowed to sediment. The sedimented starch was then washed repeatedly using distilled water accompanied with sieving after each washing until a point is reached where the wash water was clear and free of suspended impurities. The resulting starch was then sieved and dried in air at room temperature [8].

### 2.2. Preparation of Cross-Linked Enset Starch

Cross-linking reaction of enset starch was carried out following the procedures of Reddy and Seib [12] with slight modification (Table 1). Enset starch (100 g, dry basis) was suspended in distilled water (150 ml) which contains 3 g of dissolved NaCl and continuously stirred at 25°C. After adjusting to pH 10.0 with 1 M NaOH, epichlorohydrin at different concentrations (3 to 16 g per 100 g of dry starch) was added directly to the slurry for low and high level of cross-linking, respectively, with stirring at 25 to 54°C for different cross-linking time (1 to 10 h), then adjusted to 6.0–6.5 with 0.2 M HCl, and the cross-linked enset starch was isolated by centrifugation (3000 × g, 15 min). After washing with distilled water, the sediment was then dried at 45°C for 48 h in a vacuum oven (MEMMER, GmbH D-91126, Schwabach, FRG, Germany) [12].

### 2.3. Determination of Degree of Cross-Linking (DC)

Degree of cross-linking was determined using peak viscosities of the starch samples according to the methods described by Kaur et al. [13] with slight modifications. Native and cross-linked aqueous starch suspensions (10%) were prepared and heated from 50 to 90°C in water bath (D 3006, Geseltschaft fur Labortechnik mbH, Burgwedel, Germany). Their peak viscosities were measured at 50, 70, and 90°C. After keeping the samples at 90°C for 2 min, the same procedure was followed to record the peak viscosities while the samples were cooled back to 50°C and held at this temperature for 2 min. The maximum viscosities obtained during the heating and cooling process were used to calculate the DC using Equation (1) [13].

(1) $$\begin{array}{c} DC=\frac{A-B}{A}\times 100, \end{array}$$

where *A* is the peak viscosity in cP of the native starch (NS), and B is the peak viscosity of the cross-linked starch (CLS) in cP.

### 2.4. Determination of Swelling Power and Solubility

Swelling power of the NS and CLS was determined in accordance with the method described by Odeku and Picker-Freyer [14] with slight modification. A sample (0.5 g) was placed into each of the predried and weighed centrifuge tubes containing 10 ml of distilled water. Then, the suspensions were heated in a thermostated water bath (D 3006, Geseltschaft fur Labortechnik GmbH, Burgwedel, Germany) at controlled temperatures of 25, 37, 50, 65, 75, and 85°C, respectively, with frequent mixing at 5 min interval. After 30 min, the tubes were cooled and centrifuged at 3000 rpm for 15 min. The supernatant was then collected and dried in oven (MEMMER, GmbH, D-91126, Schwabach, FRG, Germany) at 130°C for 2 h. The residue obtained after drying the supernatant (*W*_1_) at a specific temperature indicates the amount of solubilized starch in water at that particular temperature and the weight obtained from the residue (*W*_2_) represents the swelling of the starch [14].

The solubility (*S*) was calculated as g per 100 g of sample on dry weight basis (*W*3) and calculated using Equations (2) and (3).

(2) $$\begin{array}{c} \text{Solubility }\left(\%\right)=\frac{W_{1}}{W}\times 100, \end{array}$$

(3) $$\begin{array}{c} \text{Swelling power }\left(\%\right)=\frac{W_{2}}{W_{3}\;\left(100-S\right)}\times 100. \end{array}$$

### 2.5. Determination of Moisture Content

Moisture contents of the CLS and NS were determined following the gravimetric (loss on drying) method mentioned in the USP-30/NF-25 (2007) for modified starches. Accordingly, 2 g of each sample was weighed into previously washed, dried, and preweighed Petri dish and heated in an oven (Kottermann®2711, H. JURGENS & CO., Bremen, Germany) at a temperature of 120°C for 4 h. The samples were then weighed, and the moisture contents were determined from the results of triplicate measurements using Equation (4) [15].

(4) $$\begin{array}{c} \text{Moisture content }\left(\%\right)=\frac{W_{1}-W_{2}}{W_{1}}\times 100, \end{array}$$

where *W*_1_ and *W*_2_ are the weights of starch samples before and after drying, respectively.

### 2.6. Determination of Moisture Sorption Property

Moisture sorption properties were determined according to the method described by Odeku and Picker-Freyer [14] with slight modification. Starch samples (5 g) were predried in an oven (Kottermann®2711, H. JURGENS & CO., Bremen, Germany) at 120°C for 4 h and were spread evenly on a predried and weighed Petri dishes and transferred to a particular relative humidity chambers that contained an appropriate solvent. The samples were then equilibrated for four weeks at room temperature. Finally, the weights after four weeks were recorded, and the moisture uptake of each sample was calculated using moisture differences of the starch samples before and after equilibration [14].

### 2.7. Fourier Transform Infrared Spectroscopy (FTIR) Studies

The structural changes due to cross-linking and the compatibility of the enset starch with theophylline were assessed using the FTIR. Finely grounded starch sample (10 mg) was mixed with mulling agent (Nujol) in a mortar and pestle. The sample mixture was then placed between potassium bromide (KBr) plates to form a thin film of the mull by compression. The sandwiched plates were placed in the IR spectrophotometer, and the spectra were obtained with 20 scans and spectral resolution of 2 cm^−1^. The scanning was performed between wave numbers of 4000 and 600 cm^−1^. The background spectrum was collected before running each sample [16].

### 2.8. Preparation of Microspheres

Cross-linked starch microspheres were prepared using the water-in-oil emulsion technique described by Hamdi et al. [17] with slight modifications. For a single batch, 5/1 ratio of aqueous to organic phase was prepared by dissolving the drug (12%, *w*/*w*) and 8 g of NES enset starch. Then, the aqueous phase was emulsified in 100 ml of cyclohexane : chloroform mixture (4 : 1 *v*/*v*) containing 2% of sorbitan monooleate (Span80). The mixture was first homogenized at 1300 rpm for 3 min using a high-speed stirrer (ISG-hotplate and magnetic stirrer) and then at 1200 rpm for 6 h at 40°C. The formed microspheres were then isolated using a suction pump filtrator and washed with cyclohexane followed by distilled water and ethanol 95% (*v*/*v*). The microspheres were then kept in a closed container [17].

### 2.9. Determination of Particle Size and Size Distribution

Particle size and size distributions of the microspheres were measured according to the sieve analysis method described by Behera et al. [18]. The microspheres were separated into different size fractions (%, weight fraction) by sieving the microspheres for 10 min in a series of standard sieves that have mesh apertures of 1000, 710, 500, 355, 250, and 180 *μ*m. Then, the distribution of microspheres were determined, and the mean particle size distributions of the microspheres were calculated using the following formula (Equation (5)) [18].

(5) $$\begin{array}{c} \text{Mean particle size}=\frac{\sum \left(\text{mean particle size of the fraction}\times \text{weight fraction}\right)}{\sum \text{w}\text{eight fraction}} \end{array}$$

### 2.10. Determination of Entrapment Efficiency

Drug entrapment efficiency is the concentration of the incorporated material (active ingredient) detected in the formulation over the initial concentration used to make the concentration. To determine the amount of drug inside the microspheres, a method described by Molla et al. [19] was used. One hundred milligrams of the prepared microspheres were weighed and crushed using a clean mortar and pestle. Accurately weighed 20 mg of powdered microspheres were added to a volumetric flask containing 100 ml of pH 6.8 phosphate buffer solution and stirred for 2 h. After 2 h, the solution was filtered, and absorbance was assayed for all formulations spectrophotometrically at 271 nm [7]. The amount of drug inside the microspheres was determined in a triplicate basis, and drug entrapment efficiency of all formulations was calculated using Equation (6) [19].

(6) $$\begin{array}{c} \text{Entrapment efficiency }\left(\%\right)=\frac{\text{Actual drug loading}}{\text{Theoretical drug loading}}\times 100. \end{array}$$

### 2.11. *In vitro* Drug Release Profile of the Microspheres

Drug release from the microspheres was analyzed using USP dissolution type-II apparatus (Pharmatest, Germany), which was adjusted to rotate at 50 rpm. The amount of microspheres equivalent to 100 mg of theophylline drug was initially dispersed into a 900 ml acidic dissolution medium (pH 1.2) for 2 h and then in phosphate buffer (pH 6.8) for the next 10 h. The temperature was maintained at 37 ± 0.5°*C*. Aliquots of 5 ml were withdrawn at time intervals of 0.25, 0.5, 1, 2, 3, 4, 6, 8, 10, and 12 h. Each withdrawn sample was replaced with equal volume of fresh dissolution medium to maintain the sink condition. After filtration and necessary dilutions, the samples were analyzed using the UV-Visible spectrometer (JENWAY, LTD. FELSTED, UK) at 271 nm [12].

### 2.12. Kinetics and Mechanism of Drug Release

The drug release mechanisms of the tablets were evaluated by fitting the *in vitro* dissolution data of the drug into different release kinetic models: zero order, first order, Higuchi square root model, Hixson-Crowell cube root models, and Korsmeyer-Peppas model. The best model was selected based on the goodness of fit test [20].

### 2.13. Optimization of Sustained Release Formulation

Based on the results of the preliminary studies, concentration of ECH and length of cross-linking time were the two independent factors that significantly affected the response variables (release rate and DC). Thus, the Design Expert® V 8.0.7.1 software (Stat-Ease Inc., Minneapolis, MN, USA CCD) was applied during the entire optimization process. Using high and low levels of the two independent factors (*n*), CCD that considers five levels for each variable [21], determined the total number of experiments to be 13 (i.e., 2^nd^ full factorials, 2*n* axial points and *n*_*c*_ center points) (Table 2). Therefore, a total of 13 experiments were carried in the optimization process.

### 2.14. Statistical Analysis

Statistical analysis was carried out using the analysis of variance (ANOVA) on a computer software package called Origin 8.0 (Origin Lab ™ Corporation, MA, USA) and a software called Design Expert® V 8.0.7.1 (Stat-Ease Inc., Minneapolis, MN, USA CCD) which helps to reveal the influence of each factor on the response variable and to point out the optimum level of factors applied to optimize the formulation. At 95% confidence interval, *p* values less than or equal to 0.05 were considered as statistically significant. All the data measured and reported in this study are averages of a minimum of triplicate measurements, and the values are expressed as mean ± standard deviation.

## 3. Results and Discussion

### 3.1. Characteristics of Cross-Linked Enset Starch for Preliminary Study

#### 3.1.1. Degree of Cross-Linking (DC)

The test result of DC (Table 3) revealed that almost all the formulations showed discrepancies in their peak viscosity and thus the DC was observed to vary between the formulations. Moreover, it was ranged from the 36.70% (F-8) to 94.20% (F-7). There was a significant difference (*p* < 0.05) in the DC among formulations of F-1, F-2, F-3, F-4, F-5, and F-6. But, differences in DC between formulations (F-1 and F-8) and (F-4 and F-7) were not significant (*p* > 0.05). Among the formulations, F-7, which was prepared using the highest values of the three factors showed relatively a maximum DC. In contrast, the formulation (F-8) which was prepared using the lowest values of all the three factors was observed to have low DC value. Likewise, except for the cross-linking temperature, increasing the amount of ECH and length of cross-linking time between the eight formulations was observed to cause a significant (*p* < 0.001) increase in the DC.

### 3.2. Swelling and Solubility Property

As it was reported by different literatures, the nature of drug release can be affected by the level of cross-linking, which in turn greatly influences the solubility and swelling property of the cross-linked starch used as a pharmaceutical excipient. In this study, the swelling and solubility properties of the native and cross-linked enset starches were measured as a function of temperature (20-85°C) [4, 19, 20].

**Figure 1:**
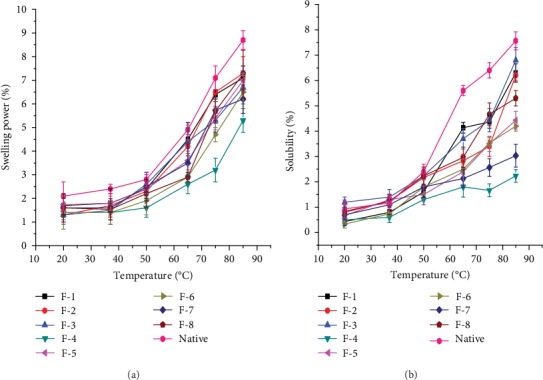
Swelling power (a) and solubility properties (b) of the native and CLS.

As it is indicated in Figure 1, there was a significant difference (*p* < 0.001) in the swelling and solubility properties of the native and cross-linked enset starches. In the native starch, both properties were observed to increase significantly with temperature as opposed to the cross-linked starches which showed no prominent changes of swelling and solubility properties as a function of temperature. This might be attributed to the strengthened and more compact bonding between starch chains due to the cross-linking process that allows them to resist against swelling and solubility [7, 10].

In addition, these properties were shown to significantly decrease (*p* < 0.05) with increasing concentration of the cross-linker (ECH) and duration of cross-linking time. Enset starch formulations (e.g., F-4 and F-7) cross-linked at high concentration of cross-linker for a prolonged time were found to show lesser swelling and solubility properties as compared to the other formulations. This could be due to the greater density of cross-links and enough time to undergo sufficient cross-linking reaction to form strong intermolecular bridges between starch granules and the cross-linker that allow them to have less disintegration property [22].

### 3.3. Moisture Content

Moisture content of the CLS formulations was determined using the difference in weight of the formulations before and after drying. As displayed in Table 3, moisture contents of the formulations were ranged from 4.2 to 15.7%.The unmodified starch was observed to contain higher moisture (15.7%) than the cross-linked enset starch formulations. Formulations with greater DC were observed to show less difference in their weight and thus low moisture content as compared to those formulations with low DC. The less moisture content of the formulations with greater DC could be attributed to the cross-linking reaction that made the starch to have more strengthened and compacted structure that can hinder the mobility of the starch granules to hold water [23].

### 3.4. Fourier Transform Infrared (FTIR) Spectra

**Figure 2:**
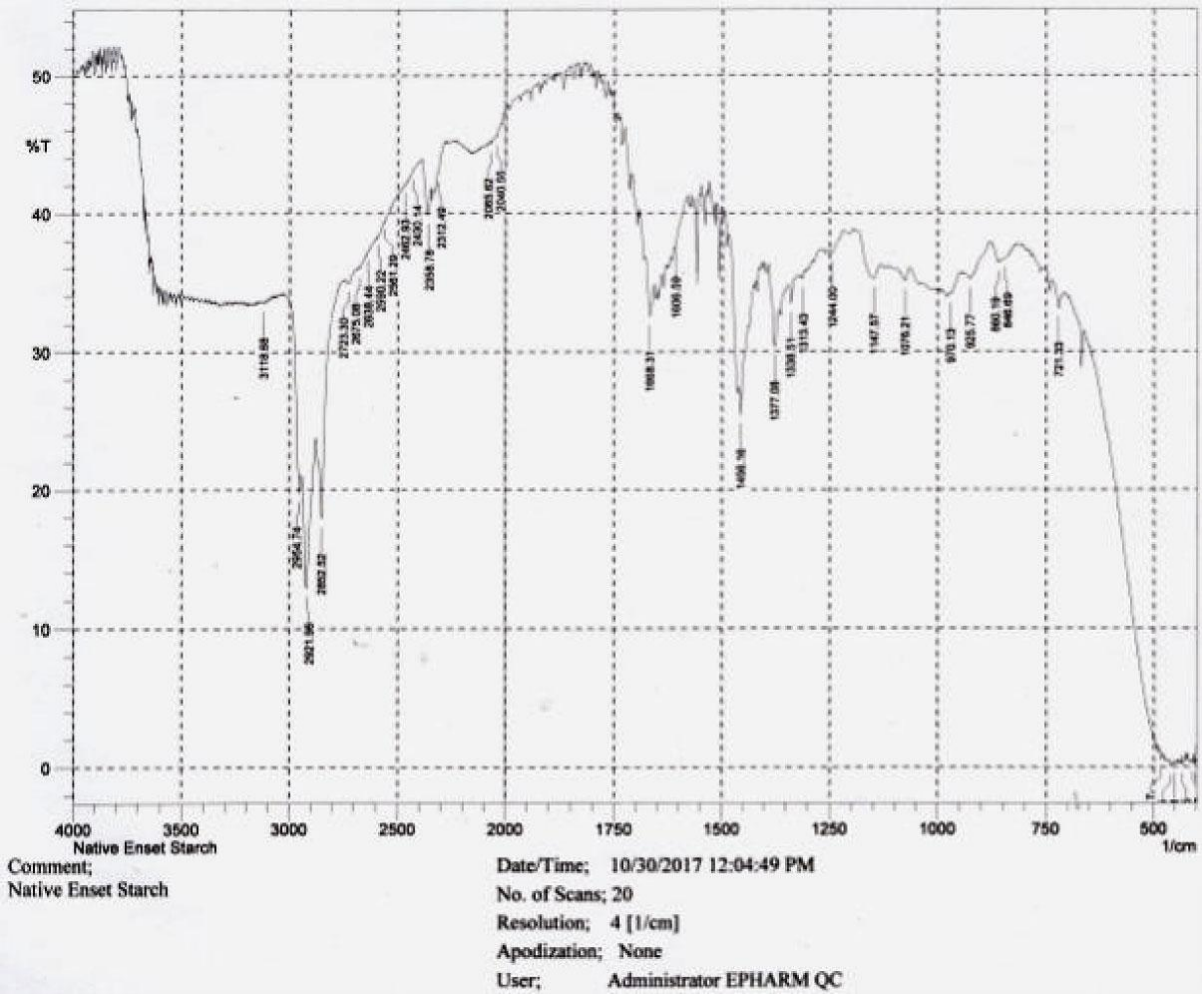
FTIR spectrum of native enset starch.

**Figure 3:**
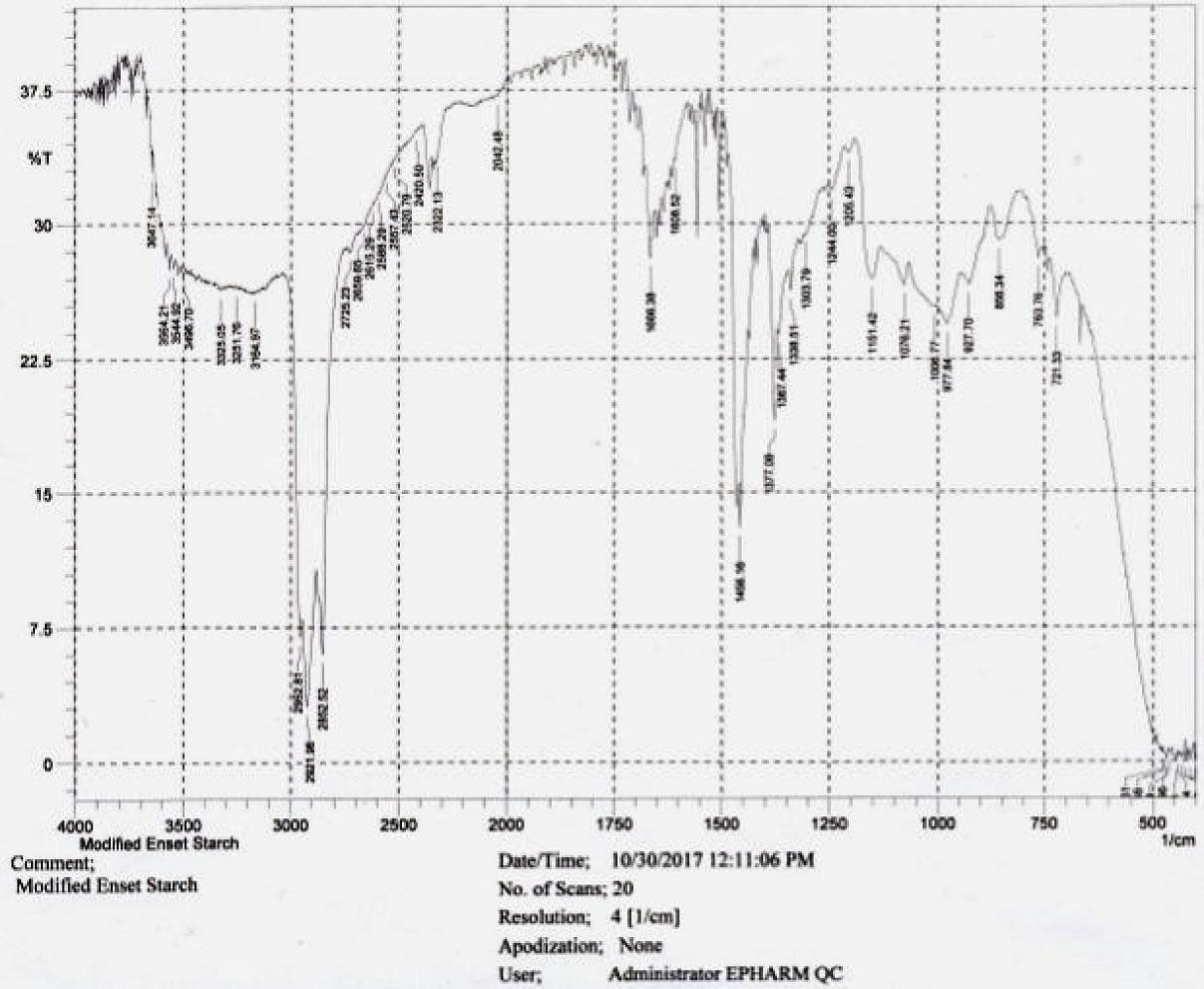
FTIR spectrum of optimized CLS enset starch.

Compatibility test of the native and optimized enset starch was assessed against the model drug (theophylline). Besides, structural change of the native enset starch due to the cross-linking reaction was confirmed using the result of the FTIR test. Figures 2 and 3 depict the FTIR results of native enset starch and optimized cross-linked enset starch, respectively. As depicted in Figure 2, the FTIR result of the native enset starch displayed all the typical absorption bands for polysaccharides, namely, a broad absorption peak around 3500-300 cm^−1^, which is due to the hydrogen bond and characteristic absorption bands for C-C/C-O stretching vibrations between 1250-1000 cm^−1^ [7, 11].

Figure 3 depicts the FTIR result of the optimized cross-linked enset starch. As can be seen from the Figure, all the characteristic bands of the polysaccharides are present in the spectral result of the optimized enset starch. In addition to all the peaks available in the native starch spectrum, the spectrum of an optimized cross-linked enset starch showed additional small new absorption bands between 1750 and 1500 cm^−1^, which confirmed the formation of carbonyl (ether) functional group (C=O) due to the cross-linking reaction. Likewise, another weak new absorption band was observed between 1250 and 1000 cm^−1^ and this might be due to the newly formed C-C/C-O stretching vibrations due to the cross-linking reaction. Furthermore, an overlapped IR spectrum of both optimized cross-linked and theophylline evidenced that the typical absorption peaks of both samples appear at the same position and wave number. Therefore, this indicated the compatibility between the mixture of theophylline and optimized CLS starch.

### 3.5. Preparation of CLS Microspheres for Preliminary Study

**Figure 4:**
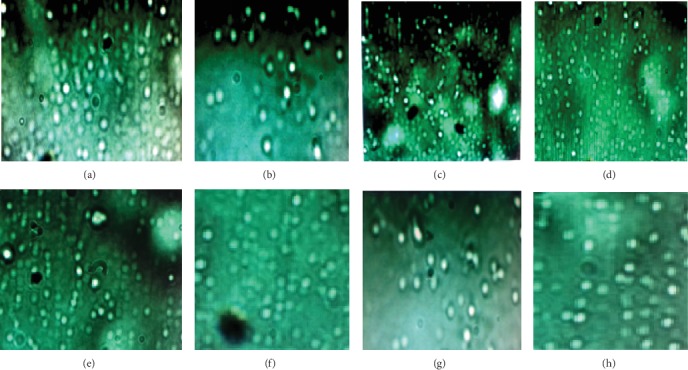
Sample microscopic pictures (100×) of enset starch microspheres formulations of F-1 (a), F-2 (b), F-3 (c), F-4 (d), F-5 (e), F-6 (f), F-7 (g), and F-8 (h) of the preliminary formulations.

The formation of the microspheres was confirmed with the help of optical microscopy (Figure 4). According to the results of the optical microscope and sieve analysis, the microspheres were spherical in shape with almost similar size range of 203.0 ± 2.8-286.0 ± 0.6 *μm*. The similarity in the size of the microspheres might be attributed mainly to the constant speed of stirring (i.e., 1200 rpm as well as to the other reaction compositions (ratio and volume of organic and aqueous phase, temperature, and span-80) which were kept constant during the preparation of the microspheres. This finding is corresponded well with previously reported findings [9, 13] that stated the greater chance of getting microparticles (microspheres) with uniform size when the stirrer speed, temperature, volume, and proportions of the organic and aqueous phase are kept constant.

### 3.6. Characteristics of CLS Microspheres

The yield and entrapment efficiencies of the prepared microspheres using cross-linked enset starch were measured as per the established procedures, and the results are presented in Table 4. Particle sizes of the cross-linked starch microspheres were ranged from 232 ± 1.1 *μm* to 273 ± 1.5 *μm*. The yield of the microspheres was ranged from 81.7 to 91.2% and the entrapment efficiency from 76.6 to 88.2%. Generally, both the yield and the entrapment efficiency were found to be higher for microspheres prepared from cross-linked starch as compared to the native one. Furthermore, these two parameters found to be higher in formulations with greater DC (F-4, F-5, F-6, and F-7). Drug entrapment efficiency is mainly influenced by cross-linking density. Formulations with better degree of cross-linking would contain more enset starch chains that are cross-bonded to each other. This cross-bonding of starch chains might develop dense matrix that would be able to hold relatively a large amount of drug as compared to their counter parts.

### 3.7. *In vitro* Drug Release Study of the Preliminary Formulations

**Figure 5:**
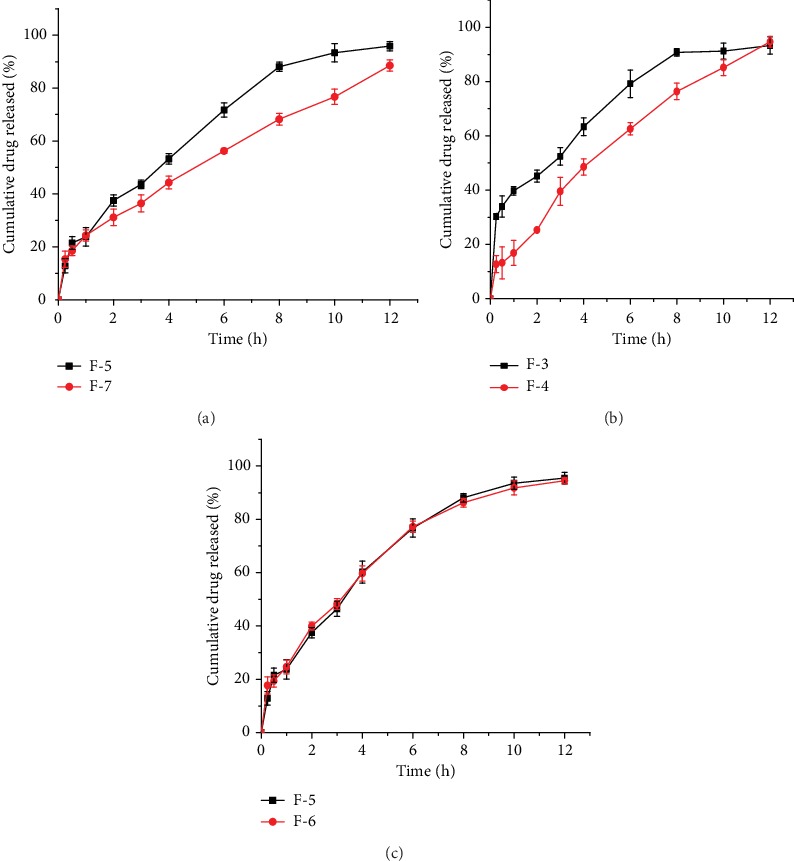
Effect of conc. of ECH (a), cross linking time (b), and cross-linking temperature (c) on cumulative release of theophylline from the microspheres.

The drug release profiles of the microspheres are illustrated in Figures 5(a)–5(c). At the early dissolution time, almost all batches of the microspheres showed a fast release of drug in both medias (acidic media pH 1.2 and phosphate buffer of pH 6.8), and this burst release could be related to the release of drugs present at the outer surface of the microspheres. Microspheres of the modified starches started to release the drug gradually. On the contrary, microspheres formulated from the native starch released 86.2% of their drug content within the first 2 h.

As it is clearly observed from the release profiles of different batches of microsphere formulations, F-1, F-2, F-3, F-5, F-6, F-7, and F-8 released more than 30% of their drug content within the first two hours. Sustained release up to 12 h was achieved in F-4, F-5, F-6, and F-7. On the other hand, formulation batches of F-5 and F-6 were shown to sustain their release up to 10 h, and other formulations (F-1, F-2, F-3, and F-8) could not hold their drug contents beyond 8 h. It was observed that among the different formulations, F-4 exhibited better extended release and released more than 90% of its drug content at the end of 12 h. Moreover, F-7 has released 31.1% of its drug in the first 2 h and released not more than 88.5% of its drug at 12 h. In relation to the other formulations, it is this formulation that has released lesser amount of drug at 12 h. The variance in release profiles of the formulations might be attributed to the poor integrity of the polymer made from low levels of the cross-linker (F-1, F-2, F-3, and F-8) and low levels of cross-linking time (F-1, F-5, F-6, and F-8) which might have contributed to the inadequately cross-linked starch and ultimately to poor integrity of the polymer to control and sustain the release of drug [24].

### 3.8. Effect of Cross-Linker Concentration

A significant decrease (*p* < 0.0001) in the drug release rate from 28.4 to 26.8 h^1/2^ and a significant increase (*p* < 0.0001) in the DC from 39.7 to 93.8% (Table 5) was observed when the concentration of the cross-linker was increased from 3 to 16 (%, *w*/*w*) (Figure 5(a)).The decrease in drug release rate and DC may be due to the fact that the higher concentration of the cross-linker leads to an increase in the number and cohesiveness of bonds in the starch which would have conferred the ability to retard the release of the drug distributed inside the starch matrix. In addition, the increase in the concentration of epichlorohydrin may bring an increase in the density of cross-links, and this may hinder the mobility of amylopectin chains and their capability of entrapping water in the matrix which in turn could slow the rate of drug release [17, 22].

### 3.9. Effect of Cross-Linking Time

It was observed that formulations that contained cross-linked starch polymer prepared from shorter cross-linking time (1 h) were not able to sustain their drug content up to 12 h. However, up on increasing the reaction time (10 h), a significant change (*p* < 0.001) of the release rate from 29.5 to 27.9 h^1/2^ and these formulations were able to delay the release of their drugs (Figure 5(b)).On the other hand, a significant increase (*p* = 0.0032) in the DC of the formulations from 62.6 to 94.2% (Table 6) was noticed upon increasing the cross-linking time. This could be partly on account of the higher reaction efficiency of the cross-linking reagent with starch chains at longer reaction times. The better reaction efficiency as a function of reaction time could be explained based on slower acting nature of epichlorohydrin. The chemical needs somewhat extended time to gradually penetrate in to the starch granules and slowly induce the cross-linking reaction [17].

### 3.10. Effect of Cross-Linking Temperature

Increasing the cross-linking temperature from 25 to 54°C reduced the drug release rate from 30.3 to 29.5 h^1/2^ of F-1 and F-8 and from 32.6 to 31.4 h^1/2^ of F-5 and F-6 (Figure 5(c)), respectively. Conversely, an increase in the DC from 61.8 to 62.6% of F-5 (Table 5) was noticed up on increasing the cross-linking temperature. This can be explained based on the fact that increasing the temperature up to a certain point is assumed to facilitate the given reaction by breaking the inherent bonds and making the native starch swell so that the functionalization process is facilitated as well as the reactive moieties in the starch become active and easily accessible for reagent [19]. However, the decrease in drug release as a function of temperature in this study was not found to be statistically significant (*p* > 0.05).

### 3.11. Optimization Study

The results of the preliminary experiments on the various modified starch based microsphere formulations indicated that the most important factors that brought statistically significant change on the response variable (drug release rate in 12 h and DC) were concentration of epichlorohydrin and length of cross-linking time. Hence, these factors were considered as the independent variables, and their effects on the characteristics of sustained release property of the microspheres were studied using RSM. The other variables, i.e., temperature and stirring rate were kept constant at 25°C and 1200 rpm, respectively. For 2 factors, CCD provided a total of 13 formulations as presented in Table 6.

**Figure 6:**
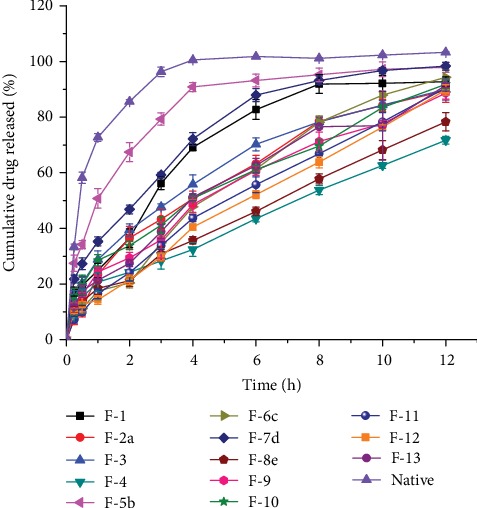
*In vitro* drug release profiles of the thirteen microsphere formulations.

As per the results of the preliminary experiments, none of the cross-linked starch microspheres were shown to have significant amount of drug release in 0.1 N HCl. Thus, all the thirteen microsphere formulations were subjected to phosphate buffer with a pH of 6.8 and their *in vitro* drug release profiles are depicted in Figure 6. As shown in the Figure, except formulations F-4, F-6c, F-8e, F-11, and F-12, most of the formulations released more than 25% of their content with in the first two hours. It was also observed that F-5b and F-7d exhibited an initial burst release of their content within the first one hour by discharging 51.1 and 35.5%, respectively. Likewise, F-5b and F-7d released much of their drug content (>90%) at the fourth (90.8%), eighth (93.2%), and third (96.3%) hours, respectively. This could be due to the fact that the starch matrices were too weak to hold their drug content when they were exposed to the dissolution medium. Which in turn might be explained on account of the less amount of cross-linker as well as short cross-linking time used during modification of the starches. On the contrary, F-4 and F-8e showed less cumulative release over the 12 h release time (i.e., 71.7% and 78.3%, respectively). This is attributed to the use of high cross-linker concentration and longer cross-linking time during the cross-linking reaction that formed more strong and compacted structure of the starch granules inside the microspheres used [17]. In addition, the increase in the amount of cross-linker particularly creates more covalently cross-linked junction zones that could increase the gel hardness and retard the release of drugs [7, 25].

Some of the formulations were found to show a significant difference in their release rate (*p* < 0.05). For a formulation to have good sustained release property, its initial (first one hour) percent cumulative drug release should be within the range of 20-25% and final (twelve hours) release of greater than 90% [13, 26]. Accordingly, from the overall drug release profiles of the thirteen microsphere formulations, it can be inferred that F-6c was observed to have better sustained release performance by releasing less than 20.4% and greater than 90% (94.3%) within the first two hours and at twelve hours, respectively.

### 3.12. Drug Release Kinetics

The drug release kinetic study results are displayed in Table 7. Except formulation F-5b, the released 90.8% of its drug content at the fourth hour (Figure 5(c)) and the remaining formulations showed the best fit for the Higuchi square root release kinetic model with higher *R*^2^ value (*R*^2^ values between 0.952 and 0.995). Hence, the Higuchi model was selected for the optimization of the release rate and DC. According to the Higuchi model for 90-100% drug release in 12 h, the release rate should be 26-30 h^1/2^ [14]. To this end, the optimization was done by targeting the drug release rate within this range and to achieve an optimum DC that can confer the best release pattern.

Table 8 depicts the drug release kinetics as per Korsmeyer-Peppas model. The Korsmeyer-Peppas equation is used to analyze the release of pharmaceuticals from polymeric dosage forms, when the release mechanism is not well known or when more than one type of the release phenomena could be involved. The mechanism is determined by *n* value, for spherical particles; “*n*” close to 0.43 indicates Fickian diffusion, *n* between 0.43 and 0.85 suggests non-Fickian (anomalous) transport, and *n* close to 0.85 shows case-II relaxation (erosion) release [27, 28]. Accordingly, *n* values of between 0.44 and 0.70 were obtained in this study, suggesting that the main release mechanism of drugs from the starch matrix of the microspheres is through non-Fickian (anomalous) transport mechanism. This is to mean that the release of drug is mainly controlled via more than one process (i.e., erosion, swelling, and diffusion) [28, 29].

In this study, optimization of the response variables (degree of cross-lining and release rate) was done by designing different microsphere formulations with the objective of getting optimum DC and the release rate of between 26 and 30 h^1/2^. As shown in Table 7, *K* value of the equation for the Higuchi square root model which denotes to drug release rate (h^1/2^) was found to be ranged between 26 and 30 h^1/2^. Furthermore, with the intention of making the release rate within the acceptable range, the range of the second response variable (degree of cross-linking) of the CLS formulations was decided to be 60-90% based on the results of the preliminary studies. This range of DC was used for further optimization process. It is clear from the Table 9 that the DC of the CLS were found to be ranged between 30.7-97.3%.

### 3.13. Selection of Mathematical Model

The Design Expert® Version 8.0.7.1 (Stat-Ease Inc., Minneapolis, MN, USA.) software was used to generate fit summaries of each mathematical model for the respective response variables. As it is clear from Table 10, the fit summary table displays different parameters namely, *p* values, multiple correlation coefficient (*R*^2^), adjusted and predicted *R*^2^, and the sum of squares (PRESS) predicted by the software. These statistical values were used to select an appropriate mathematical model (i.e., linear, factor interaction, quadratic, or cubic model) for the response variables in this study. The software by itself suggests the appropriate mathematical model based on the fit summaries of the model. Hence, the software selects a given model for the response variables provided that the model is not aliased, has adjusted *R*^2^ and predicted *R*^2^ that are in a reasonable agreement (within 0.2 of each other), and has small PRESS (sum of squares of the errors) value and a *p* value of less than or at least less than 0.1. Besides, the reliability of a given model is further supported by high *R*^2^ values [30].

In accordance to the fit summary result displayed in Table 10, quadratic model and factor interaction (2FI) were selected as adequate models for DC and drug release rate, respectively. As per the fit summary result of both response variables, the selected models were highly adequate with *R*^2^ value close to unity (>0.9), *p* value less than 0.05, small PRESS value, closely related Adj *R*^2^ and Pred *R*^2^ (within 0.2 of each other) and insignificant lack of fit *p* values of 0.3355 and 0.2497 for RR and DC, respectively.

### 3.14. Model Adequacy Checking

It is mandatory to check the fitted model to ensure that it provides an adequate approximation to the real system. Adequacy and goodness of fit of the proposed mathematical models were checked by the analysis of variance (ANOVA) at a 95% confidence interval [20].

Based on the ANOVA results in Table 11, the quadratic model for DC and the factor interaction (2FI) model for release rate were statistically significant mathematical models (*p* < 0.0001). For DC, terms *A* and *B* (the main effects of conc. of ECH and cross-linking time, respectively), *AB* (interaction effects of conc. of ECH and cross-linking time), *A*^2^ (second order effect of conc. of ECH), and *B*^2^ (second order effect of cross-linking time) were significant model terms (*p* < 0.0001, *p* = 0.0002, *p* = 0.0342, *p* = 0.0126, and *p* = 0.0100, respectively). On the other hand, for release rate, only terms A and B were the significant model terms with *p* < 0.0001 and *p* = 0.0005, respectively.

Another parameter which is helpful to judge the predictive quality of a given mathematical model is lack of fitness (LOF). LOF implies failure of the model to consider an experimental data which are not included in the regression line. A model is said to be adequate and with best goodness of fit provided that the model's lack of fit value is not significant (*p* > 0.05) [31]. Hence, as it is clear from Table 11, the LOF values of the quadratic model for DC and factor interaction for release rate were not significant (*p* = 0.2497 and *p* = 0.3355, respectively). Thus, this indicates that the models are adequate and can be used to predict the response.

Reliability of mathematical models can also be confirmed using different statistical values of the coefficient of determination such as the *R*^2^, which is expected to be greater than 0.9, the closeness between the Adj and Pred *R*^2^ (should be within 0.2 of each other), and the adequate precision (Adeq. precision), which signifies the signal to noise ratio (and a ratio greater than 4.0 is desirable).

As it is shown in Table 12, both response variables have *R*^2^ value which is greater than 0.9, reasonably agreed statistical values of Adj *R*^2^ and Pred *R*^2^ (within 0.2 of each other), and acceptable numerical value of adequate precision that indicates a good signal (i.e., 18.3 and 19.494 for DC and RR, respectively), which imply that these models can be used to navigate the design space.

From the aforementioned analyses, it can be concluded that the proposed models were considered particularly adequate to perform further analyses. The final mathematical regression models in terms of coded factors as derived by the Design Expert software were developed using model term coefficients. Therefore, the predictive mathematical models provided a regression equation (Equations (7) and (8)) in terms of coded terms of the coefficients for DC and release rate, respectively.

(7) $$\begin{array}{c} \text{DC }\left(Y1\right)=74.57+19.69\text{A}+16.92\text{B}+7.73\text{AB}-7.39A^{2}-9.27B^{2}, \end{array}$$

(8) $$\begin{array}{c} \text{Release rate }\left(Y2\right)=27.57-2.72A-1.45B-1.43AB, \end{array}$$

where *A* is concentration of ECH and B is length of cross-linking time.

Coefficients of the developed models have physical meanings on the response variables. A coefficient is the amount of the response that changes up on changing the coded terms by one unit, while keeping the other terms constant. Both the magnitude and sign of coefficients are important. The magnitude indicates the degree or strength and signs of the coefficients of regression equation that indicate the direction of change of the response variables. A positive sign indicates a positive (synergistic) effect where as a negative sign indicates a negative (antagonistic) effect on the response variables [27, 28].

In this study, as it can be observed from Equations (7) and (8), the concentration of ECH had a larger and positive effect on DC and negative effect on release rate as compared to the effect of length of cross-linking time (*B*) and their interaction term coded *AB*, which indicates increasing cross-linker concentration that increases DC and decreases release rate. This could be attributed to the tendency of ECH to make the inherent bonds of starch strong enough to increase the DC, which in turn decreases the rate of drug release [17, 22]. Therefore, it can be deduced that due to the stronger effect of term *A* on both responses, it was found to be the most critical and determinant factor for both responses. Nevertheless, the direction of the effect of term *A* was found to differ on both responses. Thus, this term was found to have antagonistic effect on release rate and synergistic effect on the DC. The same held true with regard to the direction of effect of the other coded terms on both responses variables.

### 3.15. Graphical Representations: Contour and Response Surface Plots

Graphical representations such as contour or 2-Dimensional (2D) and response surface or 3-Dimensional (3D) plots are helpful to verify the effect of each independent variable on the response variable [25]. These plots are used to visualize the point at which optimum values of the maximum or minimum response is located. Besides, the main effects as well as the interaction effects of each independent variable on the responses can be judged simply by observing the structural orientation and colors of the model graphs [27]. The curved or ellipsed nature of contour lines and the twisted shape of the 3D plot signify the interaction between the independent factors. The best predicted optimum point of the response variable is found at the area confined by the smallest ellipse of the contour plot. Likewise, colors that are graduated from blue to yellow or red implies the low and high levels of the responses with respect to the effect of the independent variable, respectively [20].

**Figure 7:**
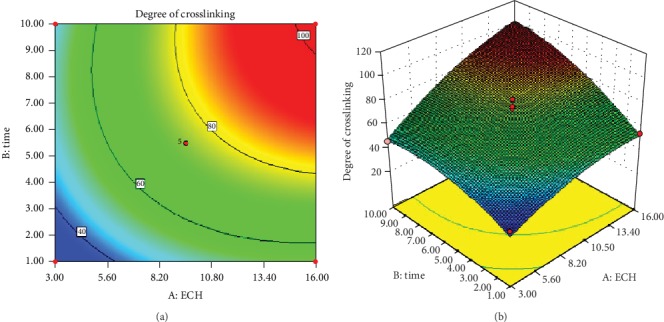
Contour (a) and response surface (b) plots of DC.

Figures 7(a) and 7(b) indicate the contour and 3D plots of DC. It was evidenced from the Figures that the curvature or ellipsed nature of the contour lines together with the twisted 3D plot indicate that the two independent factors had significant interaction effect on the DC and this was supported by the ANOVA result (*p* = 0.0342) (Table 11).

**Figure 8:**
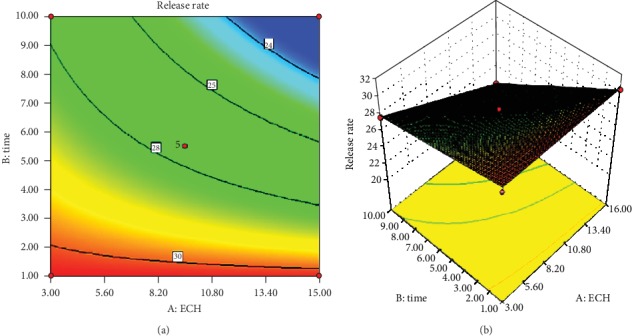
Contour (a) and response surface (b) plots of release rate.

Figures 8(a) and 8(b) show the contour and response surface plots of release rate, respectively. As it can be understood from the Figures, the contour lines were slightly curved and the 3D plot was twisted to some extent. This signifies that the two factors (concentration of ECH and length of cross-linking time) were likely to have an interaction effect. However, their interaction effect was not found to be significant. An interaction between the independent variables is characterized by the formation of elliptical contours. Similarly, the ANOVA results in Table 11 indicate that the interactive effect of the two variables was not significant (*p* = 0.0672).

### 3.16. Simultaneous Optimization of DC and Release Rate

After generating the model polynomial equations to relate the dependent and independent variables, the formulation was optimized for the two responses simultaneously. Simultaneous optimization is a model-dependent optimization technique that comprises the experimental designs, mathematical models, and graphic representations. This is an approach that is helpful to optimize more than one experimental responses concurrently. A simultaneous optimization technique attempts to provide an overall optimum value that can fulfill the objectives of each of the experimental responses [32]. Hence, by using the specific requirements or criteria for each factors and responses (Table 13), the final optimum values were obtained for both response variables using the numerical and graphical optimization techniques of the Design Expert® 8.0.7.1 software.

### 3.17. Numerical Optimization

In the simultaneous optimization approach, numerical optimization is one of the best optimization techniques that employs desirability function to provide the optimum points of the multiple responses. Desirability function is a technique that provides the way to overcome the difficulty of compromising multiple and sometimes an opposing response and searches for a mixed factor level that altogether optimizes for the response of interest [23]. Moreover, desirability function for each response can be calculated at a specified experimental range, and it ranges from zero (unacceptable value) to one (optimum value). Desirability function value that is closer to unity is preferable for a better optimum value [32]. Thus, by taking the individual desirability function values of each response into account, an overall value of the desirability function is calculated by the software solver.

**Figure 9:**
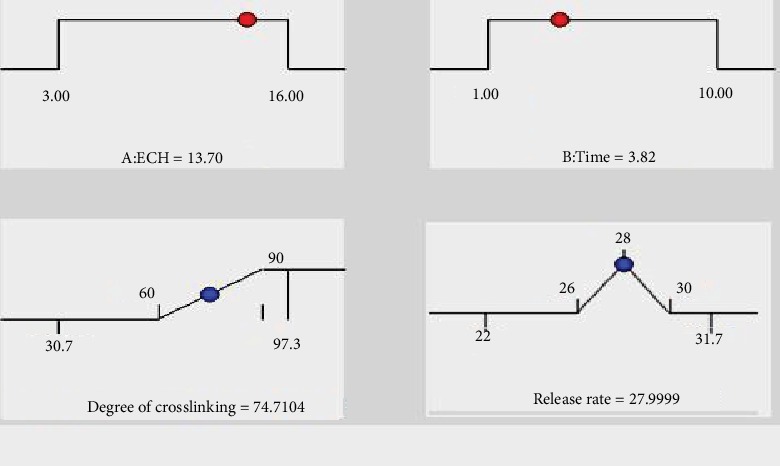
Numerical optimization results of the optimum predicted values for factors and responses.

**Figure 10:**
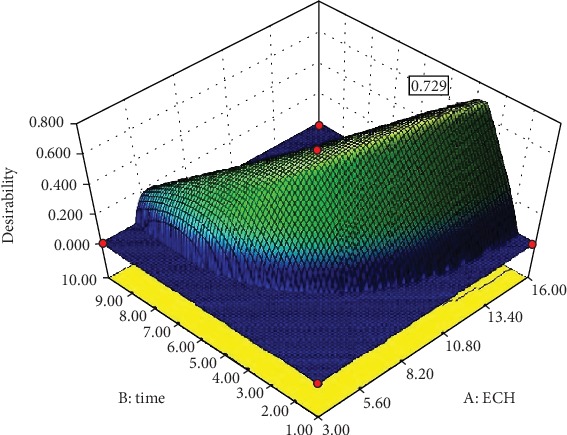
3D plot of the overall desirability function.

Figure 9 depicts the optimal point as a compromise between desired DC and release rate values. Accordingly, the partial desirability functions for both release rate and DC were found to be 1.00 and 0.490, respectively. Likewise, 0.729 (Figure 10) was the overall desirability function which was calculated by the software with the consideration of the partial desirability of both responses. The software provided an optimum point (represented by dots) and levels of factors in accordance with the specified goals (Figure 9).

### 3.18. Graphical Optimization

**Figure 11:**
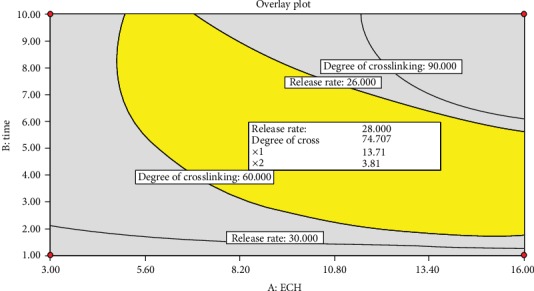
Overlay plot of release rate and DC with respect to concentration of ECH and length of cross-linking time.

Graphical optimization is the other method of optimizing multiple responses by using contour (overlay) plots to display the area of predicted optimum values for both response and factors. An overlay plot, the graphic representation for the feasible optimum points designated by a specific color (yellow), is the product of superimposed contour and response surface plots. The software solver predicts the optimum values of factors and responses that can fulfill the desired goal [27].  Figure 11 shows the area of optimum values for the factors and responses of interest. The point identified by the flag was chosen in the graph as representative of the optimized area corresponding to the ECH concentration of 13.71% and length of cross-linking time of 3.81 h. Using the specified optimum values of the factors, the software predicted the optimum values for DC and release rate to be 74.707% and 28.000 h^1/2^, respectively.

### 3.19. Confirmation Test

The results of optimum points which had been predicted by the model were verified by performing further experiments under the utilization of the provided optimum points. Thus, three batches of microsphere formulations were prepared using the optimum values of the independent variables. The three batches of CLS were tested for DC and drug release rate characteristics by preparing theophylline-loaded microspheres using the CLS as polymer. From the finding of the confirmation test, it was found that the experimental values of the optimized formulations values for both responses were corresponded well with the predicted values; the percent error is within the acceptable limit of less than 5%, which justifies the validity of the response model (Table 14). Besides, percent error of the confirmation test results for both responses were within the acceptable limit (i.e., <5%).

### 3.20. Evaluation of the Optimized Enset Starch

The optimized formulation of cross-linked enset starch microspheres formulations were further evaluated for different characteristic properties such as *in vitro* drug release profile and drug release kinetics.

### 3.21. *In vitro* Drug Release Profile

**Figure 12:**
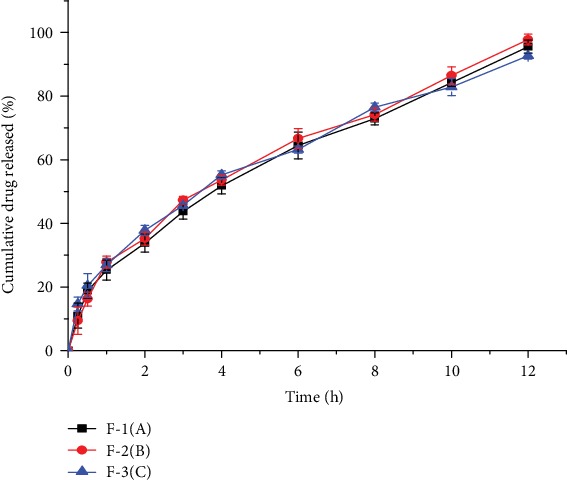
*In vitro* drug release profiles of the three batches of an optimized enset starch.

As could be verified from the *in vitro* release data of the three optimized enset starch formulations (Figure 12), there was no statistically significant difference with respect to their dissolution profile over the twelve-hour time period (*p* > 0.05). Besides, microspheres of the optimized enset starch were observed to release lesser amount of their drug content with in the first 2 h and released more than 90% (average of 93.8%) of their drug content at the twelve-hour time period and showed better sustained release pattern.

### 3.22. Drug Release Kinetics

*In vitro* drug release data of the optimized starch microspheres were fitted to the common drug release kinetic models. From the respective models, the Higuchi square root model was found to be the best-fitted model (*R*^2^ = 0.995). As per the Korsmeyer-Peppas release kinetic model analysis, the value of the exponent “*n*” was 0.526, which revealed that the drug release mechanism of the optimized enset starch microspheres was non-Fickian anomalous transport mechanism [29].

## 4. Conclusion

The cross-linked enset starch was observed to have less solubility, swelling power, moisture sorption property, and low peak viscosity (greater DC) as compared to the unmodified native counterpart. Besides, drug release property of the cross-linked starch microspheres prepared using water/oil emulsion method sustained their release up to 12 h, as opposed to the native counterparts. The preliminary studies indicated that the concentration of ECH and cross-linking time were found to be the determinant factors for the degree of cross-linking and rate of drug release properties of the microspheres prepared from cross-linked starches. Upon optimizing the factors, 13.71% concentration of ECH and 3.81 h of cross-linking time were found to be the optimal conditions. The experimental values of the theophylline-loaded microspheres prepared under the optimum conditions were in good agreement with the predicted values. The *in vitro* drug release profile of the optimum microsphere formulation exhibited minimum burst release with sustained release for 12 h. Therefore, according to the finding of this study, it can be suggested that ECH cross-linked enset starch can be used as a possible alternative drug-release-sustaining pharmaceutical excipient in microsphere formulations.

## Figures and Tables

**Table 1:** Reaction conditions of the eight cross-linked starch formulations for preliminary study.

CLS	Theophylline	ECH	1 M NaOH	NaCl	Reaction	Reaction	Weight of dry
Batch	(mg)	(%,*w*/*w*)	(%, *w*/*w*)	(%, *w*/*w*)	Time (h)	T^o^ (°C)	Enset starch (g)
F-1	100	3	6.4	3	1	54	100
F-2	100	3	6.4	3	10	54	100
F-3	100	3	6.4	3	10	25	100
F-4	100	16	6.4	3	10	25	100
F-5	100	16	6.4	3	1	54	100
F-6	100	16	6.4	3	1	25	100
F-7	100	16	6.4	3	10	54	100
F-8	100	3	6.4	3	1	25	100

**Table 2:** Experimental levels of the independent factors.

Variables	Levels				
	-*α*	-1	0	+1	+*α*
Concentration of epichlorohydrin (%, *w*/*w*)	0.31	3	9.5	16	18.69
Length of cross-linking time (h)	0.86	1	9.5	10	11.86

*α* = 1.41412.

**Table 3:** Moisture content (%) and DC (%) results of the eight cross-linked starch formulations (*n* = 3, ± SD).

Formulation code	Moisture content (%)	DC (%)
F-1	9.82 ± 3.5	37.30 ± 3.5
F-2	8.20 ± 11.3	41.55 ± 1.8
F-3	11.37 ± 4.1	39.71 ± 2.1
F-4	4.21 ± 2.1	93.80 ± 4.0
F-5	6.33 ± 1.0	62.48 ± 3.1
F-6	8.70 ± 3.7	61.81 ± 3.8
F-7	4.84 ± 4.0	94.20 ± 2.4
F-8	12.41 ± 1.4	36.70 ± 1.1
Native	15.70 ± 3.6	—

**Table 4:** Yield and entrapment efficiencies of the preliminary formulations (*n* = 3, ±SD).

Formulation	Weight of polymers before formulation (g)	Weight of polymers after formulation (g)	Yield (%)	Mean particle size (*μ*m)	Entrapment efficiency (%)
F-1	9	7.67	85.2	248 ± 2.1	78.6 ± 0.1
F-2	9	7.48	83.1	254 ± 2.0	81.2 ± 1.6
F-3	9	7.95	88.3	259 ± 0.8	79.6 ± 0.8
F-4	9	8.12	90.2	244 ± 1.1	83.6 ± 3.4
F-5	9	8.02	89.1	267 ± 0.5	80.9 ± 1.9
F-6	9	8.08	89.7	232 ± 1.1	82.5 ± 1.3
F-7	9	8.21	91.2	273 ± 1.5	88.2 ± 2.7
F-8	9	7.98	88.6	268 ± 0.3	77.3 ± 1.6
Native	9	7.36	81.7	252 ± 1.0	76.6 ± 0.7

**Table 5:** Summary of the effects of different parameters on response variable (release rate and DC).

Formulations	(ECH) (%, *w*/*w*)	Parameters and their levels		Response variable	
		Cross-linking time (h)	Reaction temperature (°C)	DC (%)	Release rate (h^1/2^)
F-1	3	1	54	37.3 ± 3.5	31.6
F-2	3	10	54	41.5 ± 1.8	28.4
F-3	3	10	25	39.7 ± 2.1	28.9
F-4	16	10	25	93.8 ± 4.0	26.8
F-5	16	1	54	62.6 ± 3.1	29.5
F-6	16	1	25	61.8 ± 3.8	30.3
F-7	16	10	54	94.2 ± 2.4	27.9
F-8	3	1	25	36.7 ± 1.1	32.4

**Table 6:** Reaction compositions for cross-linking of the thirteen formulations.

Formulations	Point type	Factors	
		Conc. of ECH (%, *w*/*w*)	Cross-linking time (h)
F-1	Factorial	3.00 (-1)	1.00 (-1)
F-2a	Factorial	16.00 (+1)	1.00 (-1)
F-3	Factorial	3.00 (-1)	10.00 (+1)
F-4	Factorial	16.00 (+1)	10.00 (+1)
F-5b	Axial	0.31 (-*α*)	5.50 (0)
F-6c	Axial	18.69 (+*α*)	5.50 (0)
F-7d	Axial	9.50 (0)	0.86 (-*α*)
F-8e	Axial	9.50 (0)	11.86 (+*α*)
F-9	Center point	9.50 (0)	5.50 (0)
F-10	Center point	9.50 (0)	5.50 (0)
F-11	Center point	9.50 (0)	5.50 (0)
F-12	Center point	9.50 (0)	5.50 (0)
F-13	Center point	9.50 (0)	5.50 (0)

**Table 7:** Rate constants and correlation coefficients of the drug release kinetic models for all the thirteen microsphere formulations.

Formulations	Release kinetic models							
	Zero-order		First-order		Higuchi square root		Hixson-Crowell	
	Slope	*R* ^2^	Slope	*R* ^2^	Slope	*R* ^2^	Slope	*R* ^2^
F-1	9.859	0.990	-0.069	0.771	29.71	0.994	-0.191	0.815
F-2a	6.652	0.887	-0.094	0.887	29.80	0.979	-0.181	0.905
F-3	6.400	0.916	-0.059	0.707	27.60	0.994	-0.188	0.830
F-4	4.628	0.854	-0.034	0.728	22.00	0.986	-0.158	0.901
F-5b	—	—	**—**	**—**	—	**—**	—	—
F-6c	6.159	0.815	-0.057	0.606	26.01	0.968	-0.168	0.842
F-7d	6.307	0.945	-0.067	0.783	31.71	0.995	-0.147	0.829
F-8e	4.207	0.987	-0.089	0.855	24.03	0.975	-0.179	0.913
F-9	6.931	0.844	-0.083	0.586	26.50	0.978	-0.126	0.928
F-10	5.069	0.782	-0.061	0.564	28.30	0.952	-0.184	0.860
F-11	4.111	0.897	-0.058	0.714	27.23	0.994	-0.118	0.931
F-12	5.965	0.881	-0.064	0.653	27.40	0.990	-0.152	0.893
F-13	6.405	0.985	-0.130	0.905	26.92	0.974	-0.128	0.851

“-”: denotes for not analyzed.

**Table 8:** The “*n*”, *K*, intercept, and *R*^2^ values of the Korsmeyer-Peppas model for the various microsphere formulations.

Formulations	*K*	Intercept	Exponent	*R* ^2^
1	0.26	-0.39	0.68	0.984
2a	0.32	-0.27	0.58	0.969
3	0.14	-0.21	0.70	0.969
4	0.25	-0.3	0.49	0.957
5b	—	—	—	—
6c	0.31	-0.38	0.44	0.934
7d	0.23	-0.54	0.48	0.989
8e	0.27	-0.36	0.43	0.961
9	0.19	-0.41	0.50	0.937
10	0.36	-0.34	0.48	0.948
11	0.25	-0.20	0.49	0.988
12	0.37	-0.56	0.47	0.979
13	0.16	-0.33	0.44	0.968

**Table 9:** Summaries of the experimental values of the microsphere formulations in terms of both actual and coded terms of the factor levels and response parameters.

Formulations	Point type	Factors		Responses	
		(ECH) (%, *w*/*w*)	Cross-linking time (h)	Degree of cross-linking (%)	Release rate (h^1/2^)
F-1	Factorial	3.00 (-1)	1.00 (-1)	33.5	29.7
F-2a	Factorial	16.00 (+1)	1.00 (-1)	55.3	29.8
F-3	Factorial	3.00 (-1)	10.00 (+1)	44.6	27.6
F-4	Factorial	16.00 (+1)	10.00 (+1)	97.3	22.0
F-5b	Axial	0.31 (-*α*)	5.50 (0)	30.7	30.3
F-6c	Axial	18.69 (+*α*)	5.50 (0)	89.4	26.0
F-7d	Axial	9.50 (0)	0.86 (-*α*)	39.5	31.7
F-8e	Axial	9.50 (0)	11.86 (+*α*)	84.6	24.0
F-9	Center point	9.50 (0)	5.50 (0)	76.4	26.5
F-10	Center point	9.50 (0)	5.50 (0)	73.5	28.3
F-11	Center point	9.50 (0)	5.50 (0)	74.6	27.2
F-12	Center point	9.50 (0)	5.50 (0)	69.3	27.4
F-13	Center point	9.50 (0)	5.50 (0)	82.7	26.9

**Table 10:** Fit summary statistics for DC (%) and drug release rate (h^1/2^).

Responses	Source	Std. dev	*R* ^2^	Adj *R*^2^	Pred *R*^2^	Lack of fit	*p* value	PRESS	Remark
	*p* value						Prob > F		
DC (%)	Linear	11.19	0.7904	0.7485	0.6439	0.0316	0.0004	2127.67	
	2FI	10.61	0.8303	0.7738	0.5928	0.0354	0.1794	2432.95	
	Quadratic	5.89	0.9594	0.9303	0.8188	0.2497	0.0067	1082.65	Suggested
	Cubic	6.69	0.9840	0.9520	0.6943	0.0375	0.2497	2230.82	Aliased

Release rate (h^1/2^)	Linear	1.16	0.8357	0.8028	0.6630	0.0891	0.0001	27.79	
	2FI	0.78	0.9342	0.9122	0.8633	0.3355	0.0052	11.28	Suggested
	Quadratic	0.86	0.9379	0.8936	0.7051	0.2051	0.8142	24.32	
	Cubic	0.67	0.9780	0.9341	0.7284	0.0373	0.2051	26.72	Aliased

**Table 11:** Summary of ANOVA results for response surface quadratic model for DC and response surface factor interaction model for drug release rate of the microsphere formulations.

Responses	Source	Sum of	df	Mean	*F* value	*p* value	Remark
	Squares		Square				
DC (%)	Model	5732.28	5	1146.46	33.06	<0.0001	Significant
	A-ECH	3101.30	1	3101.30	89.42	<0.0001	Significant
	B-time	1891.45	1	1891.45	54.54	0.0002	Significant
	AB	238.70	1	238.70	6.88	0.0342	Significant
	A^2^	384.50	1	384.50	11.09	0.0126	Significant
	B^2^	424.67	1	424.67	12.24	0.0100	Significant
	Residual	242.77	7	34.68			
	Lack of fit	147.07	3	49.02	2.05	0.2497	Insignificant
	Pure error	95.70	4	23.92			
	Cor Total	5975.05	12				

Release rate(h^1/2^)	Model	77.04	3	25.68	42.58	<0.0001	Significant
	A-ECH	52.15	1	52.15	86.48	<0.0001	Significant
	B-time	16.77	1	16.77	27.80	0.0005	Significant
	AB	8.12	1	8.12	13.47	0.0672	Insignificant
	Residual	5.43	9	0.60			
	Lack of fit	3.60	5	0.72	1.60	0.3355	Insignificant
	Pure error	1.81	4	0.45			
	Cor Total	82.47	12				

^∗^df = degree of freedom.

**Table 12:** Statistical test results of model adequacy checking for the quadratic model of DC and 2FI model of release rate.

Parameter	DC (%)	Release rate (h^1/2^)
R-squared	0.9594	0.9342
Adjusted R-squared	0.9303	0.9122
Predicted R-squared	0.8188	0.8633
Adequate precision	18.300	19.494

**Table 13:** Constraints of factors and responses for the optimization of release rate and DC of theophylline loaded CLS.

Constraints				
Factor constraints				
Factor	Low		High	
Conc. ECH (%)	3		16	
Length of cross-linking time (h)	1		10	
Response constraints				
Response	Goal	Lower limit	Upper limit	Importance
Release rate (h^1/2^)	Target = 28	26	30	+++++
Degree of cross-linking (%)	Maximize	60	90	++++

**Table 14:** Predicted optimum values, experimental confirmation test results, and percent errors for release rate and DC, (*n* = 3, ±SD).

Responses	Predicted values	Experimental values	% error
Degree of cross-linking (%)	74.70	78.31 ± 2.03	4.61
Release rate (hr^1/2^)	28.00	28.70 ± 0.90	2.43

## Data Availability

All the data's pertaining to the findings of this study are on the hands of the principal investigator. Therefore, requests for access to these data should be made to [Desta T, E-mail:dtesfay462@gmail.com].

## Abbreviations

CCD: Central composite design
CLS: Cross-linked starch
DC: Degree of cross-linking
ECH: Epichlorohydrin
FTIR: Fourier transform infrared radiation
G: Gram
HCl: Hydrochloric acid
H: Hour
KBr: Potassium bromide
M: Mole
Mg: Milligram
Min: Minute
NS: Native starch
RSM: Response surface methodology
USP: United State Pharmacopoeia.
